# Is Schizophrenia a Disorder of Consciousness? Experimental and Phenomenological Support for Anomalous Unconscious Processing

**DOI:** 10.3389/fpsyg.2017.01659

**Published:** 2017-09-28

**Authors:** Anne Giersch, Aaron L. Mishara

**Affiliations:** ^1^INSERM U1114, Pôle de Psychiatrie, Fédération de Médecine Translationnelle de Strasbourg, Centre Hospitalier Régional Universitaire of Strasbourg, Université de Strasbourg, Strasbourg, France; ^2^Department of Clinical Psychology, The Chicago School of Professional Psychology, Los Angeles, CA, United States

**Keywords:** implicit processing, unconscious processing, consciousness, schizophrenia, sensory processing, time, minimal self, self disorders

## Abstract

Decades ago, several authors have proposed that disorders in automatic processing lead to intrusive symptoms or abnormal contents in the consciousness of people with schizophrenia. However, since then, studies have mainly highlighted difficulties in patients’ conscious experiencing and processing but rarely explored how unconscious and conscious mechanisms may interact in producing this experience. We report three lines of research, focusing on the processing of spatial frequencies, unpleasant information, and time-event structure that suggest that impairments occur at both the unconscious and conscious level. We argue that focusing on unconscious, physiological and automatic processing of information in patients, while contrasting that processing with conscious processing, is a first required step before understanding how distortions or other impairments emerge at the conscious level. We then indicate that the phenomenological tradition of psychiatry supports a similar claim and provides a theoretical framework helping to understand the relationship between the impairments and clinical symptoms. We base our argument on the presence of disorders in the minimal self in patients with schizophrenia. The minimal self is tacit and non-verbal and refers to the sense of bodily presence. We argue this sense is shaped by unconscious processes, whose alteration may thus affect the feeling of being a unique individual. This justifies a focus on unconscious mechanisms and a distinction from those associated with consciousness.

## Introduction

Schizophrenia is a severe and disabling disorder affecting more than 1% of the population, and causes tremendous suffering in patients and their families. It is defined on the basis of clinical symptoms such as hallucinations, delusions, disorganization of thought, apathy and aboulia. It also includes neurobiological impairments, and cognitive disorders. Nevertheless, these latter impairments have not been integrated into the diagnosis of schizophrenia. One reason for this is that it is still unclear to what extent neurobiological and cognitive dysfunction plays a role in symptoms. A few decades ago, several authors hypothesized that schizophrenia is characterized by a failure of automatic processing leading to abnormal contents of consciousness (Frith, 1979; Maher, 1983; Venables, 1984; Gray et al., 1991). For example Gray et al. (1991) proposed that alterations in the initial stages of information processing allow items that normally remain unconscious to become conscious in patients, thus leading to abnormalities at the conscious level. Research has developed since then along these lines, yielding several models to account for the clinical symptoms of disorganization, hallucinations or delusions. Here we argue that we may deepen our understanding further by more clearly distinguishing the mechanisms associated with unconscious vs. conscious information processing. More specifically, after a brief summary of the state of the art research on these topics, we summarize recent results that compel us to look more closely at impairments at an automatic, unconscious level. We justify this proposal further on the basis of the potential links between perceptual impairments and what is termed self disorders or self-disturbances (Ichstörungen).

Cognitive impairments are usually evaluated using neuropsychological batteries, which assess memory, attention and related cognitive functions. The cognitive disorders are found to more or less preexist the clinical pathology, and persist after the onset of schizophrenia symptoms without any large variation (Becker et al., 2010; Nuechterlein et al., 2014; Bora and Murray, 2015; Metzler et al., 2015; Bergh et al., 2016; but see Zhou et al., 2017). Moreover, they predict the functional outcome of the patients (Lewandowski et al., 2013; Green, 2016; Thomas et al., 2017), but it is not clear how they relate with clinical symptoms. This leads psychiatrists to question whether cognitive disorders and clinical symptoms are independent dimensions (Bell and Mishara, 2006; Dominguez Mde et al., 2009). Despite these findings, many models have been proposed that provide indirect links between cognitive impairment and clinical symptoms such as passivity symptoms, hallucinations (Corlett et al., 2016; Jardri et al., 2016, 2017), delusions (Fletcher and Frith, 2009) and experiences of influence which includes a group of symptoms called the self-disturbances or self disorders (from the German: “Ichstörungen”) in which the conscious experience of self is affected (Franck et al., 2001; Frith, 2005; Shergill et al., 2005; Graham-Schmidt et al., 2017; Thakkar et al., 2017). We argue here that, in addition to existing theoretical models, there is a range of possible explanations which have been overlooked or forgotten. We argue that a stronger emphasis on the processing of information at an automatic level, and its relationship with mechanisms associated with consciousness, should be considered. We mainly take examples in the study of perception, for three main reasons. Perceptual disorders are observed when the pathology emerges and predict the conversion to psychosis (Mayer-Gross and Stein, 1928; Mayer-Gross, 1932; Klosterkötter et al., 2001; Bechdolf et al., 2002). They are associated with what has been called minimal self disorders (Mishara, 2007a; Martin et al., 2014; Mishara et al., 2014; Giersch and Mishara, 2017). Finally, the results of several studies suggest impairments at the automatic and unconscious levels (Laprévote et al., 2010, 2013; Lalanne et al., 2012b,c; Duval et al., 2016), which we in part review here. Perceptual and low-level sensory anomalies may thus have a stronger role in the pathophysiology and self disorders of schizophrenia than usually believed (Mayer-Gross and Stein, 1928; Mayer-Gross, 1932; Sterzer et al., 2016).

The idea that Schizophrenia is a disorder of consciousness (Frith, 1979; Anscombe, 1987) has attracted considerable interest. It is not surprising then that many subsequent studies aimed at investigating mechanisms associated with consciousness. We certainly do not question the pertinence of these studies that have expanded our knowledge considerably. In this contribution, however, we argue that on the basis of a series of recent experimental results and a long tradition in phenomenological psychopathology, the automatic, unconscious processing of sensory information may be more impaired than believed until now. We further argue that such impairments play a role in the way patients perceive their environment and themselves, an approach which in the phenomenological tradition of psychiatry has been called the “perceptual anomalies” approach (Mayer-Gross and Stein, 1926, 1928; Mayer-Gross, 1932; Uhlhaas and Mishara, 2007; Sterzer et al., 2016).

The paucity of studies examining unconscious, automatic processing in patients with schizophrenia may be due in part to initial observations that implicit measures of memory suggested that implicit memory is preserved in patients. By ‘implicit’ memory measures, we mean that instructions to subjects do not mention what is actually being measured during the task (Tulving and Schacter, 1990). For example it has been shown that exposure to words or pictures facilitate their identification at a subsequent stage (see Roediger and Challis, 1992, for an in-depth discussion on the mechanisms involved in priming). In these incidental encoding tasks patients usually benefit from the initial encoding phase to the same extent as controls (Gras-Vincendon et al., 1994). Such implicit learning seems to work well even when the type of information to be encoded is more complex (Gras-Vincendon et al., 1994; Michel et al., 1998; Danion et al., 2001b). These studies have been contrasted with those demonstrating impairments when the tasks require patients to explicitly retrieve past information (Danion et al., 2007). For example it has been shown that patients have difficulties in mentally reliving past events (Danion et al., 2007; Neumann et al., 2007), building a sense of self identity (Mishara et al., 2014; Berna et al., 2016a,b), taking prior information into account to adjust strategies (Danion et al., 2001a) or in interpreting information (Andreou et al., 2016; Moritz et al., 2016). These results and others already provide hypotheses regarding self disorders, especially self disorders as they impact patients’ life-narratives (Mishara et al., 2014; Berna et al., 2016a,b). This has led some authors to generalize these results by inferring that the mechanisms associated with consciousness are selectively altered in patients (Passerieux et al., 1995; Besche-Richard et al., 1999). A recent meta-analysis has confirmed that priming is only minimally impaired in patients with schizophrenia, with the exception of priming associated with conceptual processes (Spataro et al., 2016).

The above results have been more recently extended to non-conscious processing. As a matter of fact implicit processing, as defined in the above cited studies, refers to the instructions that are given to the subjects, but do not necessarily mean that the information that is implicitly encoded is unconscious. In implicit memory tasks, information is in fact displayed above threshold and is consciously processed and identified. In contrast to the above studies, Del Cul et al. (2006, see also Charles et al., 2017) compared priming effects when primes were detectable vs. undetectable. As the memory studies described above, the patients were impaired at explicitly reporting information, even though this information influenced their performance at a non-conscious level in the same way as the healthy controls. These results once again suggested that patients mainly have a difficulty with mechanisms associated with consciousness.

In summary, the studies reviewed above indicate that patients have difficulty with accessing information consciously. This confirms the hypothesis that mechanisms associated with consciousness are impaired in patients with schizophrenia. Still this account remains only partial, and we ask whether there is impetus to study the implicit unconscious processing by using other means. As a matter of fact, similar results have been observed in patients with multiple sclerosis regarding conscious access (Reuter et al., 2007, 2009), suggesting the difficulty in accessing information consciously is shared by several pathologies. This would fit with the idea that cognitive disorders are independent from the specific symptomatology associated with schizophrenia (Dominguez Mde et al., 2009). We report here that several studies suggest that there are perceptual impairments that remain largely unexplored in the studies we cited above.

We present studies below that indicate that spatio-temporal processing may be distorted in schizophrenia patients on an implicit, non-conscious level. One reason why we focus on perceptual experiences in patients is related to the reports of the patients themselves. As a matter of fact, perceptual abnormalities or distortions have often been described by patients, and especially during the prodromal and early stages of schizophrenia (Mayer-Gross and Stein, 1928; Mayer-Gross, 1932; Conrad, 1958). In fact, perceptual abnormalities in subjects at high risk for psychosis are one of the main predictors of conversion to schizophrenia (Klosterkötter et al., 2001; Bechdolf et al., 2002). These and other descriptions suggest that patients perceptual experience the world is distorted in these early stages. Moreover, these distorted perceptual experiences are related to an altered sense of self (Mishara, 2007a; Martin et al., 2014; Mishara et al., 2014; Giersch and Mishara, 2017). We refer here to the bodily self, or minimal self, which is the feeling of being present here and now (Gallagher, 2000; Stanghellini, 2009; Nelson et al., 2013). Nevertheless, we contend that the minimal self need not be consciously processed or experienced to play a role in how conscious experience is shaped (Mishara, 2010a, 2012). It does, however, define or demarcate self relative to one’s world. This requirement does not seem to be fulfilled in patients, who report a loss of boundaries between themselves and the external world, a sense of depersonalization, and out-of body experiences (Parnas and Handest, 2003).

The following patients self-reports, extracted from Chapman’s (1966), illustrate the distortions described by patients:

“I have to put things together in my head. If I look at my watch I see the watch, watchstrap, face, hands and so on, then I have got to put them together to get it into one piece.”

“It’s all right if it’s just one thing at a time but I am virtually blind at these times and can’t move properly because there are so many things coming into my eyes that I don’t know what’s what. I’m like a robot that somebody else can work but I can’t work myself. I know what to do but I can’t do it. When I’m in this state of confusion I can’t relate past experience to what is happening now. I can’t keep things in mind long enough.”

“When you feel in a trance, you tend to identify yourself with the other person, but that does not matter for if he moves you go back into a trance. You are dying from moment to moment and living from moment to moment and you’re different each time. You don’t know you’re in it. When I look at somebody my own personality is in danger. I am undergoing a transformation and myself is beginning to disappear.”

If clinical descriptions suggest perceptual distortions in prodromal and later stages, what do the experimental approaches tell us? As a rule, patients have difficulties in detecting and reporting sensory signals. For example, when the visibility of a target stimulus is lowered with a mask, patients need a longer delay between target and mask to detect the target (Saccuzzo and Braff, 1981; Green and Walker, 1986; Rund, 1993; Cadenhead et al., 1998; Butler et al., 2001; Tam and Liu, 2004; Koelkebeck et al., 2005). When a stimulus is flickering, the patients require a lower flickering frequency than controls to detect the flicker (Schwartz and Winstead, 1988). Even in the absence of any manipulation, patients need a higher stimulus contrast than controls to detect the presence of a stimulus. Some of these results have been questioned, especially concerning the effects of medication (Keri et al., 2002; Sheremata and Chen, 2004), methodology (Skottun and Skoyles, 2007) or the causal role of alterations at the retinal level (Silverstein and Rosen, 2015). More details on paradigms used to explore sensory processing can be found in **Table 1**.

**Table 1:** List of some lines of research aimed at testing early sensory processing impairments.

Exploration method	Some (not exhaustive) references	Targeted mechanisms	Comments
Prepulse inhibition (PPI)/accoustic startle: Reduction of the startle reflex when the stimulus inducing the startle (the pulse) is preceded by a ‘prepulse,’ i.e., a weaker sensory stimulus. The time separation between the pulse can be shorter than 100 ms.	Swerdlow et al., 2006; Li et al., 2009; Scholes and Martin-Iverson, 2010; Kohl et al., 2013; Javitt and Freedman, 2015; Morales-Muñoz et al., 2016; Vargas et al., 2016	The inhibition of the startle reflex is supposed to be automatic, especially for short delays between prepulse and pulse (<50 ms).	– The inhibition might be under attentional control (Scholes and Martin-Iverson, 2010).
		This inhibition is reduced in patients with schizophrenia. The results are consistent with the hypothesis of abnormal and excessive sensory responses.	– The lack of prepulse inhibition is not specific to schizophrenia (Kohl et al., 2013).
			– Interestingly, the reduction of the startle reflex is more marked for intervals <100 ms in patients with schizophrenia (Swerdlow et al., 2006) and in first episode psychosis (Morales-Muñoz et al., 2016). A relationship with timing needs checking.
Sensory gating: inhibition of repeated stimuli. Two clicks are displayed with an interval of 500 ms, and the electroencephalographic response to the second stimulus is reduced relative to the first one.	Bodatsch et al., 2015; Cromwell and Atchley, 2015; Javitt and Freedman, 2015; Morales-Muñoz et al., 2016	Like for the PPI, the inhibition of the response to the second click is reduced in patients with schizophrenia. It is likewise consistent with the hypothesis of abnormal and excessive sensory responses.	– The gating might be sensitive to cognitive and emotional influences (Cromwell and Atchley, 2015).
Latent inhibition (LI): The initial exposure to a stimulus prevents this stimulus to be associated with an aversive signal during a subsequent conditioning procedure.	Gray et al., 1991; Lubow, 2005; Meyer and Louilot, 2014; Vargas et al., 2016	The conditioning is not reduced in patients.	LI might represent a model of selective attention and memory rather than of early sensory abnormalities (Lubow, 2005; Meyer and Louilot, 2014).
Unconscious perceptual priming: the exposure to a stimulus that is made invisible nonetheless influences the processing of a subsequent stimulus.	Del Cul et al., 2006; Jahshan et al., 2012; Kiefer et al., 2013; Langdon et al., 2013	The unconscious perceptual priming seems to be preserved, like perceptual priming in general.	Interestingly, Kiefer et al. (2013) suggested that the time course of perceptual priming differs between patients with schizophrenia and controls.
Backward masking: the visibility of a visual target information is decreased by a ‘mask,’ i.e., another visual stimulus, when it is displayed right after the target information, in the location or close to the location of the target.	Saccuzzo and Braff, 1981; Green and Walker, 1986; Rund, 1993; Cadenhead et al., 1998; Butler et al., 2001; Schechter et al., 2003; Herzog et al., 2004; Tam and Liu, 2004; Koelkebeck et al., 2005; Granholm et al., 2009; Jahshan et al., 2012; Lalanne et al., 2012a	The patients with schizophrenia are more sensitive than controls to the effect of the mask and their perception of the target is altered. The explanations for the cause of this effect have been various (magnocellular deficit, reentrant processing).	– The results on unconscious priming, as well as those of Herzog et al. (2004) suggest that masking impairs access to the conscious perception of the prime, but leaves early information processing unimpaired, thus questioning whether these tasks reveal early processing impairments (Jahshan et al., 2012).
			– The results of Granholm et al. (2009); and Lalanne et al. (2012a) suggested that impairments in masking might be related to difficulties in orienting attention in time on the target.


*We prioritized paradigms that have been related to sensory processing and include unconscious processing or at least implicit processing (i.e., processing that does not follow an explicit instruction). The only case including pure unconscious processing is unconscious perceptual priming. In all other cases, the information is clearly perceptible and can be consciously perceived and/or anticipated. For example, even if conditioning is a non-conscious mechanism, the fact that information leading to conditioning is perceived consciously makes it possible that the processing of conditioning information is modulated by mechanisms associated with consciousness.*

Despite some gaps in our knowledge concerning the existence and mechanisms of sensory processing, the results suggest that patients with schizophrenia have dim vision in the chronic stage. Interestingly, the patients also have difficulty at grouping visual information (Silverstein and Keane, 2011). Recognizing forms requires several processing stages, among which grouping plays an important role. In the primary visual cortex, distinct populations of specialized neurons allow for the extraction of primitives like orientation, luminance or color. These neurons have small receptive fields, meaning that visual information is first processed locally and in parallel. Information belonging to the same figure must thus be bound together and distinguished from information belonging to different figures or to background. These binding processes are necessary to derive and identify object forms (Boucart et al., 1994). This entails several iterative processes, however, because visual information is most of the time ambiguous. The ambiguity can result from various factors, e.g., dim light, view angle variability, or occlusion by other objects in the foreground. The resolution of this ambiguity is impaired in those patients (Silverstein, 2016; Silverstein et al., 2017), who have significant difficulty in exploring visual information (Obayashi et al., 2003; Minassian et al., 2005; van Assche and Giersch, 2011). Interestingly, many of the experiments exploring these difficulties relied on the incidental role of grouping factors. For example, grouping factors like collinearity have been manipulated, since this factor, i.e., the alignment of contour elements, is one of the strongest grouping factors (Boucart et al., 1994; Kovács et al., 1999; Giersch et al., 2000; Shipley and Kellman, 2003). The results showed that patients are impaired at detecting forms defined by collinear elements (Silverstein and Keane, 2011). Moreover, many experiments were designed to show an improvement of performance in patients relative to controls, thus allowing the authors to exclude a non-specific generalized deficit as the cause of the patients’ impairments. Already in the seminal studies, Place and Gilmore (1980) and Wells and Leventhal (1984) showed that patients with schizophrenia did not benefit from the organization of lines when counting them, even though this was the easiest condition for controls. Disorders in organization mechanisms in patients also allowed them to be faster than controls in a visual search task, when compared to a control condition without grouping cues (van Assche and Giersch, 2011). The possible link between these impairments and perceptual reports of patients, or symptoms, has been conceptualized within the framework of two main models. One is related to the idea that the contextual modulation of neuronal firing is impaired in patients (Phillips et al., 2015; Silverstein, 2016; Silverstein et al., 2017). This impedes patients from refining information processing to disambiguate visual signals. As a matter of fact, although we have usually no difficulty to identify forms, visual information is inherently ambiguous. For example when forms are partially hidden by foreground objects, the visible parts can be interpreted in different ways, e.g., as being separated or as belonging to the same object. Ambiguous information would initially result in one piece of information corresponding to many possible solutions (e.g., objects). Iterative processing and the progressive integration of additional information would help to discard possibilities and finally reach a more accurate interpretation of the information in healthy subjects. This would involve the modulation of neural processing at the local level and would be impaired in patients with schizophrenia. Dim vision and false interpretations might then result in bizarre perceptions, and even delusions (Uhlhaas and Mishara, 2007).

Another conceptual model is related to predictive coding (Friston, 2005, 2008). This model also involves iterative processing, but this time each possibility issued from information processing would correspond to an hypothesis, or prediction, which is compared to the sensory input to check if it matches or not. Once objects are identified, the same mechanisms can be used to compare the visual input from moment to moment and to detect changes. If information stays the same, it is considered as non-pertinent and canceled, whereas a detection of a change is considered as pertinent and attracts attention. Many studies have suggested these mechanisms are impaired in schizophrenia (Gray et al., 1991; Friston and Frith, 2015; Sterzer et al., 2016; Corlett, 2017; Griffin and Fletcher, 2017). This impairment results in a difficulty to detect changes in perceptual information. This might lead to something similar as contextual modulation, i.e., dim vision. Both models explain bizarre perception by the emergence of aberrant information, related to a confusion between pertinent and non-pertinent information, or to erratic contextual modulation or grouping. Why and when aberrant contextual grouping or prediction errors occur is unclear, however. Patients with schizophrenia are not always impaired at grouping stimuli (van Assche and Giersch, 2011). They can sometimes use prediction errors efficiently (Delevoye-Turrell et al., 2002, 2003; Knoblich et al., 2004). It can even happen that they suffer more from unpredictability and irregularity than from regularity (Horacek et al., 2016), as if they would be more rather than less sensitive to change. These inconsistencies suggest that a closer look at the mechanisms underlying these impairments is warranted. If one were merely to consider the literature reviewed at the beginning of our contribution, then it is easy to see how such findings could have been related to the hypothesis of impaired conscious processing relative to more intact automatic, unconscious processing. Recent results challenge this assumption, however, and suggest that impairments at the automatic and unconscious levels should be explored more closely. They may help to reconcile some inconsistencies in the literature.

## First Series of Results: Low Spatial Frequency Processing

The first series of results concern contrast sensitivity. The evaluation of contrast sensitivity consists in displaying sinusoidal gratings, i.e., stimuli composed of alternating black and white bands, the contrast of which varies according to a sinusoidal law. Subjects are asked to detect the presence of the grating. The manipulation of the contrast between dark and light bars makes the grating more or less visible, and allows for the evaluation of a detection threshold. This threshold varies according to the spatial frequency composition of the gratings. Low spatial frequencies convey global information and correspond to dark and white bars that are distant in space, whereas high spatial frequencies convey detail information and correspond to dark and white bars that are close in space. As noted above, results usually suggest that patients with schizophrenia have decreased contrast sensitivity. This deficit is usually more marked when the stimuli are composed of low spatial frequencies (Butler et al., 2001; Jahshan et al., 2017). However, studies exploring the perception of spatial frequencies in an incidental way found the opposite result. Laprévote et al. (2010, 2013) first filtered stimuli, and obtained thus pictures with either high or low spatial frequencies. The authors then created hybrid pictures, which are composed of one stimulus with low spatial frequencies and one with high spatial frequencies (Schyns and Oliva, 1994, 1999). They applied this procedure to faces, while the high and low spatial frequencies faces composing the stimulus had different expressions. With this procedure, the type of expression that is perceived by the subject tells which spatial frequencies he/she perceives preferentially. This paradigm was applied to patients with schizophrenia, and the results show, in two distinct studies (Laprévote et al., 2010, 2013), that patients preferentially perceive the low spatial frequency contents. This might suggest that results observed with incidental procedures do not necessarily match those observed when patients are more directly asked to make a perceptual judgment. This hypothesis remains to be checked, though, especially as the results for conscious and unconscious processing were obtained in different groups of patients. This objection does not hold for the two following examples, though, which were obtained within the same groups of patients.

## Second Series of Results: EEG Responses to Unpleasant Stimulations

Patients with schizophrenia are frequently thought to have reduced sensitivity to pain and to emotional stimuli generally, which has been thought to be related to anhedonia (Kring and Moran, 2008). For emotion, however, recent studies rather suggest that patients experience emotions in a similar manner to controls, but cannot control them (Kring and Moran, 2008; Cohen and Minor, 2010; Horan et al., 2013). As with perception, a majority of studies relies on the subjective report of patients. Nonetheless, the results suggest that patients report emotional experiences similarly to those reported by healthy controls (Kring and Moran, 2008). Some studies also use physiological measures of emotional experiences, e.g., skin conductance or heart beat (Venables and Wing, 1962; Kring and Neale, 1996; Hempel et al., 2005, 2007). Although results are somewhat mixed a number of results have suggested that physiological responses may be even larger in amplitude in patients with schizophrenia than in controls (Kring and Moran, 2008). Here again there might be a contrast between the automatic physiological response to emotion, and the subjective report. Such a contrast has also been found with unpleasant, electrical stimulations. There is less studies on pain than on emotion, and here again most of them rely on the subjective responses of the subjects after a stimulation. We will not review the data helping to determine which mechanisms associated to pain processing are impaired in patients with schizophrenia (see, e.g., Bonnot et al., 2009; Lévesque et al., 2012; Engels et al., 2014; Stubbs et al., 2015), but will focus on one study suggesting heightened responses at a non-conscious level when no alteration is evidenced at a conscious level. Duval et al. (2016) used both electrical stimulations right below pain threshold and pictures with a negative emotional content. Subjects were asked to rate the intensity and unpleasant character of the stimuli subjectively, while EEG was recorded continuously. There was no difference between groups on subjective ratings, neither for electrical stimulations nor for pictures with a negative emotional content. However, EEG recording showed heightened physiological responses for both types of stimuli. Patients displayed early EEG responses 50 ms after electrical stimulations, whose amplitude was larger than in controls. EEG responses after pictures with an emotional content were also heightened in patients, at around 1 s after the picture display. The results as a whole again suggest that automatic responses can be altered in patients with schizophrenia without this leading to observable abnormalities at a conscious level. Moreover these alterations at an automatic level are not necessarily congruent with disorders at a conscious level. As a matter of fact heightened sensitivity to pain is rarely observed in patients with schizophrenia (but see Girard et al., 2011). All in all these abnormalities remind us of the heightened sensitivity to sensory information in subjects at risk of developing schizophrenia (Mayer-Gross and Stein, 1928; Mayer-Gross, 1932; Klosterkötter et al., 2001; Bechdolf et al., 2002). This heightened sensitivity would, however, be observable only at an unconscious level in patients with chronic schizophrenia. The third example is also consistent with this hypothesis.

## Third Series of Results: Time Processing

We have seen in the first example that patients have difficulties in organizing visual information in space, with an attendant fragmentation of information. Phenomenological approaches lead to similar observations about time (Fuchs, 2007, 2013; Vogeley and Kupke, 2007; Mishara, 2010b). Experimental psychology methods confirm that conscious experience is structured in time (van Wassenhove, 2009; Wittmann, 2011; Elliott and Giersch, 2016). Information that occurs within windows of 20–50 ms (in monosensory conditions) is judged to be simultaneous, and temporal simultaneity can help to group information together (Kandil and Fahle, 2004). On the other hand, asynchrony would help to distinguish successive events from one another, and to order them. Perceived asynchronies would thus be fundamental for organizing events in time. The succession of events and the relationships that are established between events also contribute to the sense of time continuity, which has been reported to be altered in patients with schizophrenia (Fuchs, 2007, 2013; Vogeley and Kupke, 2007). Given the potential importance of time event-structure in the ability to organize events in time and the sense of time continuity, it seemed clinically relevant to check the ability of patients with schizophrenia to distinguish sensory stimuli in time. In these studies, two visual squares are typically shown on a computer screen, each in a different location, and subjects determine whether the two stimuli are simultaneous or asynchronous (analog studies are also conducted in the auditory modality). Subjects give their response by pressing on one side in case the stimuli are simultaneous, and on the other side in case they are asynchronous. Several studies have now established that patients are impaired in time discrimination, and need larger delays between stimuli to distinguish them in time (Foucher et al., 2007; Giersch et al., 2009; Schmidt et al., 2011). In addition, these results have been shown to be independent of trivial effects such as an attention or bias effect (Giersch et al., 2009; Lalanne et al., 2012c). What is especially relevant regarding the present argumentation, however, is the contrast between this difficulty at a conscious level, and the impairments evidenced at a non-conscious level.

**FIGURE 1:**
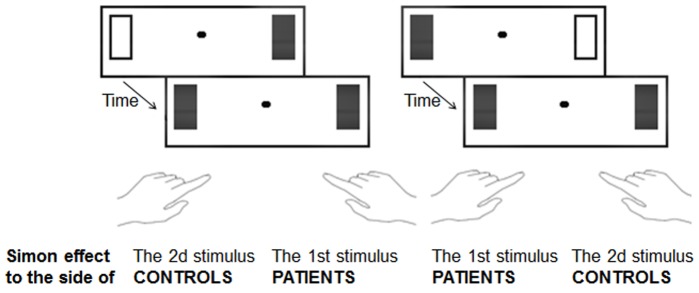
Illustration of Simon effect used to evaluate the non-conscious processing of information in time. The two stimuli displayed on the screen were separated by a variable stimulus onset asynchrony. Subjects decided whether the two stimuli were simultaneous or asynchronous and gave their response on the left side or on the right accordingly. When asynchronies were shorter than 20 ms and simultaneity ratings were identical to those observed witth synchronous stimuli, healthy subjects nonetheless tended to press to the side of the 2nd occuring square, whereas patients tended to press to the side of the 1st square.

We have indeed repeatedly shown that even when two stimuli are judged to be simultaneous, they are nonetheless automatically distinguished and followed in time (Lalanne et al., 2012b,c; Poncelet and Giersch, 2015). The Simon effect can be used to indicate that this automatic processing has taken place. The Simon effect refers to the fact that subjects tend to answer on the side of the stimulus whatever the task demands may require (Simon, 1969). For example, if subjects have to discriminate forms, e.g., circles and squares, and if they are instructed to press on the right side for circles, they will nonetheless tend to press on the left side if the circle is displayed on the left side. When two stimuli are shown on the screen, as in the simultaneity/asynchrony discrimination task, the Simon effect cannot be applied directly. If information is perfectly symmetrical on the screen, there cannot be any Simon effect. An asymmetry can be observed in case of an asynchrony, though, and such a temporal asymmetry has indeed been shown to induce a Simon-like effect (Lalanne et al., 2012b,c). Healthy subjects were shown to have a bias to press to the side of the second square in all cases, and this effect was interpreted subsequently as indexing the ability of subjects to follow stimuli over time. As a matter of fact, by the time the subjects gave their response, the attention of the subjects was on the location of the last stimulus (see Poncelet and Giersch, 2015 for more detailed experimental evidence). Patients with schizophrenia were shown to have biases similar to controls when the asynchronies were visible (i.e., above 50 ms). The critical results were observed when asynchronies were not perceptible. Patients showed a Simon effect even for asynchronies as short as 8 and 17 ms (Giersch et al., 2015). This shows that they distinguish information in time better than what was suggested from their subjective responses. Most importantly, however, for the present discussion, the Simon effect was opposite to the one evidenced in the controls. Whereas controls tended to press to the side of the second square even for stimuli separated by a short delay (i.e., 17 ms), the patients tended to press to the side of the first stimulus (**Figure 1**). Hence, in contrast with healthy subjects, who would be able to follow stimuli over time and displace their attention toward the most recently occurring stimulus, patients appeared to be “stuck” with the first stimulus.

It was argued that these results reveal a difficulty of the patients with schizophrenia to anticipate and follow information over time smoothly. Anticipation was evoked due to the very short delay between stimuli onsets (Giersch et al., 2016). This short asynchrony makes it difficult for subjects to initiate an attention displacement from the first to second stimuli once the first stimulus has been displayed. The first stimulus requires some time no matter how brief to be processed (Scharnowski et al., 2009; Herzog et al., 2016). There is thus a risk that the onset of the second stimulus is missed. To avoid this risk, the two stimuli can be attended for in advance. This is likely the case during an experiment in which subjects are instructed to judge about the simultaneity or asynchrony of the two stimuli on each trial. More recent data confirms the fragility of time anticipation in patients with schizophrenia (Giersch et al., 2017; Martin et al., 2017). The fact that anticipation mechanisms are altered in patients with schizophrenia can explain why they appear to be stuck with the first stimulus and is consistent with phenomenological hypotheses (Martin et al., 2014; Giersch and Mishara, 2017), as developed below. In this third example, patients with schizophrenia once again display impairments at a non-conscious, automatic level that differ from the impairments at the conscious level.

Since these impairments affect mechanisms that are not accessible to consciousness or introspection, it may be tempting to simply dismiss them. It is thus legitimate to ask how the impairments affect the conscious experience of the patients. In doing so, we should be reminded how much unconscious mechanisms shape our experience of the world. Above, we briefly described the mechanisms involved in the recognition of objects. Most of these mechanisms are unconscious. We are not aware that specialized information is extracted at the level of the primary visual cortex, and that this information has to be bound together in order for objects to be identified. Nonetheless, an impairment of these mechanisms can lead to visual agnosia, whereby patients have difficulties recognizing objects. It can be stressed here that patients with visual agnosia are unable to clearly convey what they see. For example, HJA is a patient who suffered from visual agnosia (Giersch et al., 2000). Extensive exploration allowed researchers to infer that he suffered from an impairment in binding information together (Humphreys and Riddoch, 1987). However, despite coming regularly to the laboratory and receiving extensive information about the results of the tests, HJA was unable to describe what he saw. This means that if patients with schizophrenia suffer from an impairment of the unconscious mechanisms associated with anticipation and temporal structure-event coding, it is likely that they are unable to clearly describe what they experience. Moreover, a difficulty in organizing information in time properly can be expected to disrupt subjective experience. The dialog with phenomenology is helpful here to conceptualize how impairments at an automatic level can disrupt the conscious experience of patients. In the following section, we give a brief example of how impairments in the automatic processing of information in time might fit with and complement existing models of perceptual impairments in patients with schizophrenia and phenomenological analysis.

## Impairments of Time-Event Structure Coding At An Automatic and At A Subjective Level: Phenomenological and Clinical Meaning

The impairments described above are not inconsistent with the models cited above, i.e., neural modulation or predictive coding. We have seen that fragmentation in space has been related with clinical disorganization, and this might be also the case for fragmentation in time. Besides it has been argued that the anticipation of future events is congruent with predictive coding (Lalanne et al., 2012b; Tschacher et al., 2017). The models of neural modulation or predictive coding can be applied at any level of processing, both unconscious and conscious. Yet, this should not prevent us from distinguishing unconscious and conscious processing. The heightened weight of low spatial frequencies, the abnormal increase of the physiological responses in case of an electrical stimulation, or the high temporal accuracy at a non-conscious level, contrast with dim or blurred responses at a conscious level. The heightened responses and the fragmentation of information processing at a non-conscious level may reflect alterations starting from the onset of information processing. These alterations might distort the perceptual experience at its core, and induce a strangeness or other anomalous experiences in conscious experience that might be difficult to report.

It is when trying to derive the consequences of disorders in the automatic processing of information on the subjective experience of patients that the dialog with phenomenology can contribute. We illustrate this point with the example of time impairment. We have proposed elsewhere that time impairments might play a more fundamental role in the structure of consciousness. As already stressed by philosophers (Husserl, 1991), time continuity is inherent to subjective experience. Time flows and seems resistant to any efforts to stop it. The fact that there are no *experienced* gaps in our subjective experience of time continuity makes such continuity seem to be a given fact, which we generally do not question. Moreover, the obligatory flow time as irreducible ‘fact’ contributes to our sense of being a unique individual, who experiences continuity over time. Conversely, the disruption of the feeling of continuity might impair the feeling of being one unique individual. This disruption has often been described at a phenomenological level in patients with schizophrenia (Fuchs, 2007, 2013; Vogeley and Kupke, 2007). We have argued that impairments in time discrimination at an automatic level might contribute to the disruption of time continuity and thus result in disturbances of minimal self (Martin et al., 2014; Giersch and Mishara, 2017). Importantly, we argue that it might be misleading to focus on the impairments at the subjectively conscious level while ignoring those at the automatic level. A difficulty in predicting and following information at an unconscious level at the very brief time scales described above may represent an elementary mechanism at the root of the sense of time continuity. This sense is too robust to rely exclusively on the ability to consciously relate events with one another. We have proposed that the automatic mechanisms enabling events to be both distinguished and followed over time may play a major role in the sense of time continuity. It follows that the non-conscious impairments in temporal organization in the patients should alter the patients’ experiences. In patients, an excess of information would be processed at an unconscious level without being integrated in the flow of consciousness, and would contribute to the strangeness or other anomalous experiences of the conscious experience in patients. Such an impact of unconscious mechanisms on the conscious experience has also been explored in phenomenology, and supports this hypothesis. The phenomenological approach on this question is briefly summarized in the following section. This will enable us to go back to the experimental data now informed by the phenomenology and not only discuss how it leads to an adaptation of existing models but also to new questions.

## The Phenomenological Approach to Schizophrenia Suggests An Implicit Timing Deficit

Founded by the mathematician turned philosopher, Edmund Husserl (1859–1938), phenomenology is the rigorous, methodical description of conscious experience. Phenomenology can be applied to study general mental structures in human experience and how these are disrupted in neuropsychiatric disorders. It is well-known that phenomenology studies consciousness, i.e., what appears as given in consciousness. What is less well-known is that it also studies the unconscious automatic processing which plays a decisive role in the organization of conscious experience (Mishara, 1990; Wiggins, 1994; Wiggins and Spitzer, 1997). As such, phenomenology stands in an excellent position to bring the experimental data concerning the disruption of implicit processing in schizophrenia together with the often bizarre symptoms of psychosis, such as the self-disturbances or self disorders (from the German: “Ichstörungen”). It is beyond the scope of the present paper to discuss the current debates in phenomenology on how minimal self is best characterized and how it is accessed by phenomenological method (Mishara, 2007a,b, 2010b; Zahavi, 2007; Parnas et al., 2008; Mishara et al., 2014; Sterzer et al., 2016). What we will discuss here is how disorders in the temporal organization of unconscious and conscious processing can alter a sense of minimal self, and to which extent phenomenology and experimental psychology provide arguments in favor of this hypothesis.

Giersch et al. (2013) propose that a basic disturbance in temporal coding may play a role in the perceptual anomalies, e.g., visual perception, in schizophrenia. Here the phenomenological approach to automatic processing may bring together: (1) the experimental data of patients with schizophrenia who are unable to follow events in time at an implicit level, (2) the extended temporal windows of what is experienced as simultaneous and the patient’s experience of fragmentation of the normal flow of events (Giersch and Mishara, 2017).

The phenomenological psychiatrist, Binswanger (1957) made use of the phenomenological approach to study automatic processing (called genetic phenomenology) in his analyses of self and schizophrenia. He proposed that psychosis itself is a “natural experiment” in which “deeper,” otherwise inaccessible unconscious levels of processing are exposed. The phenomenology of automatic processing involves the systematic removing of phases of complete-objects in their constitutive meaning, starting with the most conscious ones first – as if peeling the layers of an onion – to see what layers remain underneath. These layers, or genetic phases become separated ‘abstractly’ in reflection, but may not be said to ‘exist’ on their own. Husserl (1966) described the implicit automatic processes of passive synthesis as a self-organizing system (Mishara, 2012).

Another group of phenomenological psychiatrists in the early 20th century, the early Heidelberg School (Gruhle, Mayer-Gross, Beringer) first named and defined the self-disturbances (from German: “Ichstörungen”) and used mescaline as a model-psychosis in healthy individuals to explore the possible mechanisms of the self-disturbances (Mishara et al., 2016; Sterzer et al., 2016). This approach anticipated Hemsley and Gray’s observation of a failure to make use of stored material. As indicated above, Gray et al. (1991) and Hemsley (1994) proposed that the “core cognitive disturbance” of schizophrenia may be an inability to make use of stored material in the processing of current input. Gray (1998) observes there is an “excess of conscious, controlled processing relative to normal individuals” (Gray, 1998, p. 259) due to a failure of redundant information to become automatic which impedes the goal directedness of a limited capacity and slower controlled processing system.

The Heidelberg group observed that when healthy individuals were experiencing mescaline intoxication, they reported experiences very close to the psychotic patients. Their relationship to past experiences was profoundly modified: the past did not seem to contribute to shaping present experience and goals. One participant stated, “Everything which I see is different, isolated, without any relationship to what has happened in the past.” Another states: “I experience increasing difficulty to implement my impulses into goals and movement.” That is, the current experience is so new and compelling, there is a loss of relationship to past and future. Another subject states: “During the experiment, I happened to receive a letter of considerable importance and opportunity. I read it with complete indifference without feeling or reaction. The whole thing appeared to me to be meaningless, as if it belonged to some past time” (Mishara et al., 2016).

Moreover, the Heidelberg findings suggested novel underlying mechanisms for the bizarre psychotic experiences of the self-disturbances, where perceptions, movements, cognitions, emotions occur independently from the self’s volition. Perceiving, moving, speaking, thinking, feeling and willing are normally supported by unconscious, automatic processes, which are thus largely ignored. In the self-disturbances, automatic processes are dissociated from self, and thus experienced as not under the patient’s control (Mishara et al., 2016; Sterzer et al., 2016). This does not mean that the automatic processing itself becomes conscious. This is especially the case when the automatic processing involved occurs on such small time scales that it cannot possibly become conscious as in the experiments described above.

In the self-disturbances of schizophrenia, where one’s own automatic processing appears to function independently from self or its volition, there would rather be a disconnection between conscious experience of self and automatic processing. This means the automatic processing is experienced as acting on its own which is reported as very disturbing for patients with self-disturbances (Uhlhaas and Mishara, 2007; Mishara et al., 2016; Sterzer et al., 2016). In other words, *the psychotic experience of self-disturbances may rest on abnormalities of the coordination of timing between conscious and non-conscious processing*. The disconnection of automatic processing from attendant conscious experience can then be experienced as intrusions in the sense that phenomena enter consciousness due to the lack of coordination in time between automatic and conscious controlled processing. For example thought insertion, thought withdrawal, experiences of influence in all domains (perceiving, moving, speaking, thinking, feeling, and willing), appear as being detached from the patient’s conscious controlled processing. This experienced lack of control makes the patients own embodied processing seem alien and influenced by alien agency.

Mayer-Gross is the herald of what later came to be known as the “perceptual anomalies” approach to the positive symptoms of schizophrenia, the view that low-level perceptual anomalies play a critical role in the positive symptoms of schizophrenia, including the self-disturbances. The phenomenological psychiatrists Matussek, Conrad and Binswanger later developed this view (for reviews see Uhlhaas and Mishara, 2007; Mishara, 2012; Mishara and Fusar-Poli, 2013). In his phenomenological analysis of the loss of common sense in schizophrenia, Blankenburg arrives at similar conclusions. As Blankenburg (1969/2001) observes, our mental health is preserved thanks to a certain “resistance” to losing our common sense. This resistance functions precisely by ignoring what is obvious, which does not require further exploration; it even “resists” further exploration. But once this resistance is lost, its absence is painfully salient. A patient states, “What is it that I am missing? It is something so small, but strange, it is something so important. It is impossible to live without it…”. Blankenburg (1969/2001) continues, “Not only is the patient pained to have lost something which seems to be very small but she must also suffer that the rest of us barely appreciate this essential component of our experience and have the greatest difficulty to empathize with what it would be like not to have it.” Blankenburg (1969/2001) writes, “The healthiness of common sense rests on habituality. The natural self-evidence of everyday existence draws its nourishment from just such a habituality.”

Blankenburg observes that common sense rests on judgments of the probable rather than what we can directly ascertain as true. If one demands certitude in the proper domain of the merely probable (as in Blankenburg’ s patient attempting to select a dress for a special occasion), then one attempts to construct with controlled effortful processing what is available to others as a matter of “subtlety of feeling, i.e., what is intersubjectively acceptable but not accessible to direct rational analysis…”. This is consistent with the fact that automatic processing is impaired and must be compensated for by conscious processing. Moreover it shows how the disconnection between unconscious and conscious processing may lead to the strange self-reports of patients, and contribute to disturbances of the minimal self.

Earlier, we suggested that the minimal self (the non-verbal sense of own bodily presence) is compromised in schizophrenia. The minimal self is in part defined by temporal structure which need not be conscious. Moreover, the role of temporal structure in the experience of bodily presence (one’s own and others’) is shaped by unconscious processes, whose alteration may thus affect the feeling of being a unique individual.

This is suggested by Husserl’s own phenomenological analyses of the experience of time. In these analyses, Husserl observed a fundamental paradox in how we experience time. Time is both “streaming” (the experience of subjective flow) and “standing” (we are always in a current now). Husserl was particularly disturbed by this paradox, and found it to be a central enigma not only to how we experience time but also self. How could the experience of subjective time be composed of two apparently contradictory elements: a streaming and a standing in the streaming? Yet without these two elements there would be no experience of time?

The German phenomenological philosopher, Held (1966) comments on Husserl’s dilemma: “it is possible to lead the enigma of the living present back to a fundamental problem: the non-experiencability of the full unity of standing and streaming” (our translation). Citing Husserl: “The functioning I leads continuously into its own horizons of the past and future because it itself is what in the transitioning remains standing” (Das ich fungiere leitet in sich kontinuerlich in seine Vergangenheits- und Zukunftshoirzonte ueber, weil es selbst in stehen uebergaengig ist).

Husserl identifies the standing in the streaming as the self: The standing (persistent, self-transcending) “I” continuously emerges as invariant structure of time: the streaming itself. The I is the temporal structure of streaming (that it is a totality which I do not simultaneously grasp but only live as a “letting be” of the flowing itself -“urpassive Enstroemen-Lassen”-). That is the self only preserves itself by the letting be of the obligatory flow to which it is subjected. The originally passive streaming (i.e., without participation of the I) is given in advance in a manner that remains puzzling [raetselhafte Vorgebenheit des urpassiven Stroemens (Held, 1966, p. 102)]. But just as the passive, obligatory streaming of time seems given in advance so is the self or I as what stands or remains the same in the streaming. Held (1966) cites a manuscript by Husserl: The stream is always ahead but the I is also ahead (Das Stroemen ist immerzu im Voraus; aber auch das Ich ist im Voraus) (Ms. C 17, IV S 5ff, 1932, our translation). That is, the streaming can only be experienced by an «I» who somehow remains preserved. Conversely, the I only preserves itself by letting itself go in an ongoing process of self-displacement, a giving way to its own division (Entzweiung) in which the current I recalls the just past I as now its double in an ongoing process of self-regeneration. At the center of the self’s nunc stans is a submitting to its own transcending.

Following Mayer-Gross and Stein (1928) and Mayer-Gross’ (1932) view that low-level perceptual anomalies play a critical role in generating the positive symptoms of schizophrenia, including the self-disturbances, we suggest here that anomalies in very early stages in the temporal ordering of sensory processing already at very brief time scales (as examined in the above experiments) may also contribute to the self-disturbances and related symptoms. Such anomalies could disrupt the experience of self in time. The I as what is preserved in the momentary transitions from moment to moment is based on the original unity between standing and streaming of the living present now (Held, 1966). To the extent that one or the other of the mutually interdependent components standing and streaming is disrupted it will lead to a disruption of its complement. This very much fits in with early Heidelberg School (Beringer, 1927; Mayer-Gross and Stein, 1928; Mayer-Gross, 1932) phenomenological study of the model psychoses of the self-disturbances (Mishara et al., 2016; Sterzer et al., 2016). For example, the Heidelberg participants reported “Movements are experienced as abnormally slow or not seen at all: the movement of my hand is only experienced at its beginning and end positions. Conversely, movements are exaggerated, seen as abnormally fast, resting objects are seen as moving. My movements appear to me as artificial, foreign, like an automaton.” With regard to the distortions of time and space in the model psychoses of the self-disturbances, subjects reported: “I could not envision future or past. I lived entirely in the present, and even that in an entirely thin slice. Time slows down, coming to a complete standstill with a sense of timelessness, or conversely, starts to speed up. When time stands still, there is loss of sense of movement and space” (Mishara et al., 2016). We have examined elsewhere how these phenomenological findings are consistent to predictive coding accounts of positive symptoms in schizophrenia, including the self-disturbances (Sterzer et al., 2016; Giersch and Mishara, 2017).

## Integrating Experimental and Phenomenological Approaches: Toward A New Model?

**FIGURE 2:**
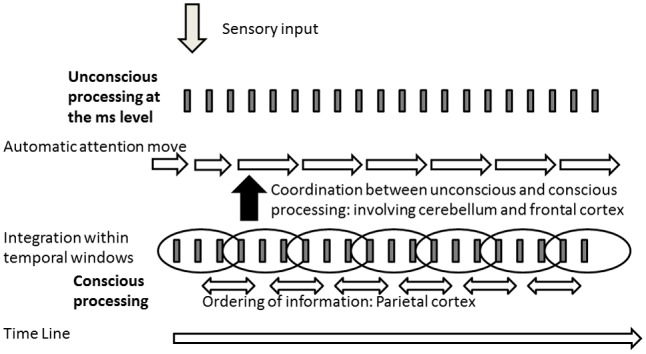
Illustration of the model derived from the hypothesis of a dissociation between the processing of information at the unconscious and conscious levels. Information would be ordered only at the conscious level (involving the parietal cortex –Battelli et al., 2007; Davis et al., 2009; Binder, 2015), requiring a time coordination between the processing at the unconscious and conscious levels. Inasmuch this coordination would involve predictive mechanisms at the level of the ms, it points toward an interaction between cerebellum and the frontal cortex (Andreasen, 1999; Giersch et al., 2016).

The phenomenological approach emphasizes both the disconnection but also the relationship between unconscious and conscious processing in an emergent self-organizing field. The dissociation is also supported by the experimental data. In the following, we illustrate why this dissociation is important and should encourage more careful examination and experiments to examine unconscious processing and its relationship with conscious processing. We develop this point by speculating on the possible consequences of the dissociation based on timing data. It is often assumed that the main difference between conscious and unconscious levels is the existence or lack of conscious realization. Models of consciousness are indeed a way to resolve the question of consciousness access (Baars, 1989; Crick and Koch, 1995; Tononi and Edelman, 1998; Lamme and Roelfsema, 2000; Changeux and Dehaene, 2008). However, many unconscious mechanisms never reach consciousness. Moreover, our data suggest that some mechanisms of integration may be selectively associated with consciousness, thus implying a qualitative change in consciousness relative to the unconscious level. In other words we have to understand not only how information becomes conscious, but also what additional information or factors enter at the conscious relative to the unconscious levels. In particular, we have argued elsewhere (Giersch et al., 2013) that it is unlikely that all information is automatically compared and ordered relative to one another in time, due to a problem of exponential combinatory cost in crowded environments. In addition, it can be questioned if the Simon effect observed at sub-threshold asynchronies reflects a processing of time order (inasmuch it reflects a bias to give a motor response to the side of the second event in a sequence of two). If it was, we would have expected that the order of the stimuli (right-left or left-right) is encoded even when presented at subthreshold asynchronies (<20 ms). This should have facilitated the processing of a subsequent sequence of stimuli when these stimuli are presented in the same order (for example 1st sequence right-left and 2nd right-left). This was not the case, however (Poncelet and Giersch, 2015). On the contrary the processing of the 2nd sequence of stimuli was facilitated when it was in the opposite direction relative to the 1st sequence. The Simon effect thus allows us to attend to the last occurring information without necessarily coding the order relationship between successive information. The Simon effect appears to reflect a mechanism allowing us to stay connected with the flow of information rather than to order information. If this is true, ordering information might be a property selectively associated with consciousness. It follows that the dissociation between unconscious and conscious mechanisms would require coordination between these mechanisms. The ability to predict and follow information more accurately at the unconscious than the conscious level would help to embed unconscious mechanisms within conscious temporal sequences. Reversely, the processing of order associated with consciousness might be used to adjust automatic and unconscious mechanisms so that they fit together in time. For example, when the aim is to orient one’s attention, automatic mechanisms have to fit in time with the attention sequence. This hypothesis is illustrated in **Figure 2**. The impairment of this interaction might explain the dissociation between unconscious and conscious processing described in phenomenology in patients with schizophrenia. Such a hypothesis may reconcile the models of contextual modulation and predictive coding, by introducing the question of temporal coherence between different levels of processing. The contextual modulation would require a temporal alignment of automatic sensory mechanisms on the temporal ordering sequences at a conscious level. If automatic and conscious processing are not coordinated, it may contribute to defective contextual modulation and blurred or dim vision. It may also imply that the content of sensory information cannot be properly compared to prior expectations. As a matter of fact, time coordination may require the prediction of sequences of sensory events in advance in order to coordinate all the mechanisms involved in information processing. We have already proposed that prediction mechanisms at the ms level are impaired in patients with schizophrenia (Giersch et al., 2016), which may be consistent with the cognitive dysmetria hypothesis of Andreasen (1999). This impairment might result in items not being properly integrated in time, and thus emerging abnormally at the conscious level, as proposed by Gray (1998). However, the function of the prediction we propose here differs from the other models (without excluding them). Its main function would be to time and coordinate unconscious and conscious mechanisms, rather than including an expectation regarding the content of the sensory information. It remains to be seen whether the neurophysiological mechanisms associated with consciousness are involved in this coordination, which would enable further integration with neuronal connectivity and associated genetic results in this model (Uhlhaas and Singer, 2010; Balu and Coyle, 2011; Gurung and Prata, 2015; Martino et al., 2017; Romme et al., 2017). A link between neurobiological impairments and minimal self disorders has already been proposed by some authors (Mishara et al., 2016; Northoff and Duncan, 2016; Northoff, 2017). Our proposal here adds possible explanations at the functional level.

All in all, this proposal is not incompatible with previous models, but provides testable hypotheses on specific timing mechanisms. Its originality is to contrast unconscious and conscious processing. If it is confirmed that some mechanisms are specifically associated with conscious processing, it will require us to re-consider how information is processed unconsciously and how it is coordinated with conscious processing. This might shed new light on the patients’ impairments, as tentatively developed here.

## Conclusion

In summary, recent experimental evidence suggests contrasting impairments of unconscious and conscious sensory processing. The heightened responses to unpleasant information, or the abnormalities in time processing, contrast with the perception at the conscious level, which is noisy and dim. This contrast may contribute to a disconnection between automatic and conscious processing. In this sense the results help to reconnect with the hypotheses that had been put forward years ago, and that explain the loss of natural evidence that characterize the distorted experience of patients (Blankenburg, 1971, Blankenburg, 1969/2001) and may underlie their self-disturbances.

## Author Contributions

AG wrote the first draft of the manuscript and revised it. AM wrote the part on phenomenology and revised the manuscript.

## Conflict of Interest Statement

The authors declare that the research was conducted in the absence of any commercial or financial relationships that could be construed as a potential conflict of interest.
